# THE CORONAVIRUS JOB RETENTION SCHEME AND MENTAL HEALTH AMONG OLDER WORKERS: EVIDENCE FROM ENGLAND

**DOI:** 10.1093/geroni/igac059.764

**Published:** 2022-12-20

**Authors:** Giorgio Di Gessa, Paola Zaninotto

**Affiliations:** UCL, London, England, United Kingdom; UCL, London, England, United Kingdom

## Abstract

The COVID-19 pandemic has led to major economic disruptions. In March 2020, the UK implemented the Coronavirus Job Retention Scheme –known as furlough –to minimize the impact of job losses. So far, little is known on the mental health impact of this scheme on older workers, and on whether this varies by job characteristics. Exploiting longitudinal data from Wave 9 (2018/19) and two COVID-19 sub-studies (June/July 2020; November/December 2020) of the English Longitudinal Study of Ageing we use logistic and linear regression models to investigate associations between changes of employment and mental health during the pandemic. About 10% of respondents aged 52-67 were furloughed in the initial phase of the pandemic. Overall, employment disruption was associated with changes in mental health, although results suggest differences by pre-pandemic job characteristics (i.e. hours worked, physical effort, social class, and stress measured by the effort-reward imbalance model).

